# Supplementary material for: Using sero-epidemiology to monitor disparities in vaccination and infection with SARS-CoV-2
Source: Nat Commun. 2022 May 4;13:2451. doi: 10.1038/s41467-022-30051-x (PMC9068757; doi:10.1038/s41467-022-30051-x)
Supplement: Supplementary file 1 — Supplementary Information [file 41467_2022_30051_MOESM1_ESM.pdf]

Supplementary Figures and Tables for ‘Using sero-epidemiology to monitor disparities in vaccination and infection with SARS-CoV-2’

Supplementary Tables

Supplementary Table 1: Interpretation of bivariate antibody results on the Vitros and Roche assays. Table showing the sample sizes and results of each antibody assay, including samples removed due to false positive/negative or inconclusive results.

Vitros result	Roche result	Interpretation for bivariate analyses (n)
Positive	Positive	Infected and (either vaccinated or unvaccinated) (n=79)
Positive	Negative	Uninfected and vaccinated (n=181)
Negative	Negative	Uninfected and unvaccinated (n=655)
Negative	Positive	False negative on Vitros and/or false positive on Roche (n=3; removed)
Positive	No result	(n=15; removed)
Negative	No result	(n=43; removed)
No result	Positive	(n=2; removed)
No result	Negative	(n=21; removed)

Supplementary Table 2: Bivariate posteriors by ZIP code Tabulation Area (ZCTA). Table showing the mean posterior probability of vaccination, the mean posterior probability of prior infection, and the 2.5%, 50% and 97.5% quantiles of the posterior for both prior infection and vaccination as well as sample size, stratified by zipcode.

ZCTA	Mean, P(vaccination)	Mean, P(infection)	2.5%, P(vaccination)	2.5%, P(infection)	50%, P(vaccination)	50%, P(infection)	97.5%, P(vaccination)	97.5%, P(infection)	N samples
**94102**	0.166	0.124	0.073	0.048	0.164	0.121	0.273	0.224	36
**94103**	0.097	0.169	0.025	0.079	0.091	0.166	0.203	0.278	31
**94105**	0.060	0.166	0.002	0.044	0.046	0.158	0.193	0.333	13
**94107**	0.156	0.059	0.066	0.008	0.152	0.053	0.266	0.147	28
**94108**	0.180	0.057	0.056	0.001	0.171	0.044	0.349	0.186	12
**94109**	0.257	0.101	0.178	0.046	0.256	0.099	0.341	0.170	65
**94110**	0.198	0.178	0.123	0.108	0.198	0.176	0.279	0.256	75
**94112**	0.219	0.145	0.145	0.087	0.218	0.143	0.298	0.212	86
**94114**	0.198	0.070	0.105	0.017	0.195	0.065	0.307	0.153	38
**94115**	0.359	0.023	0.258	0.001	0.357	0.016	0.467	0.080	39
**94116**	0.135	0.056	0.066	0.014	0.132	0.051	0.219	0.120	48
**94117**	0.177	0.042	0.097	0.006	0.175	0.037	0.273	0.108	41
**94118**	0.217	0.041	0.131	0.005	0.215	0.036	0.313	0.107	43
**94121**	0.315	0.052	0.229	0.012	0.313	0.048	0.407	0.115	53
**94122**	0.292	0.058	0.210	0.017	0.291	0.055	0.382	0.118	65
**94123**	0.197	0.038	0.086	0.001	0.192	0.028	0.331	0.126	21
**94124**	0.075	0.155	0.019	0.077	0.070	0.153	0.163	0.254	40
**94127**	0.322	0.097	0.166	0.015	0.317	0.088	0.494	0.235	17
**94131**	0.357	0.031	0.232	0.001	0.355	0.022	0.491	0.111	28
**94132**	0.299	0.162	0.185	0.073	0.298	0.159	0.421	0.266	40
**94133**	0.215	0.067	0.106	0.011	0.211	0.060	0.348	0.161	25
**94134**	0.257	0.231	0.136	0.130	0.255	0.227	0.392	0.347	36

Supplementary Table 3: Univariate posteriors by demography. Table showing the univariate mean and the 2.5%,50% and 97.5% quantiles of the posteriors for the assay results, as well as the raw count of positive samples and denominator, stratified by demographic group.

Assay	Demographic Group	Median	CI.2.5%	CI.97.5%	N positive (raw)	N samples with results
Roche	0-17	0.451	0.240	0.681	8	20
Vitros	0-17	0.518	0.291	0.767	9	21
Roche	18-34	0.173	0.116	0.243	23	149
Vitros	18-34	0.277	0.202	0.364	35	151
Roche	35-64	0.101	0.074	0.136	38	412
Vitros	35-64	0.223	0.180	0.271	79	426
Roche	65+	0.043	0.024	0.070	14	359
Vitros	65+	0.479	0.420	0.539	151	377
Roche	Asian	0.035	0.015	0.065	8	262
Vitros	Asian	0.331	0.269	0.399	75	272
Roche	Black or African American	0.086	0.038	0.157	8	108
Vitros	Black or African American	0.201	0.127	0.289	18	110
Roche	Hispanic or Latino	0.321	0.250	0.399	47	163
Vitros	Hispanic or Latino	0.463	0.380	0.551	67	173
Roche	Other	0.102	0.042	0.186	7	71
Vitros	Other	0.313	0.204	0.444	19	71
Roche	White	0.046	0.026	0.072	14	337
Vitros	White	0.328	0.270	0.387	96	350

Supplementary Table 4: Bivariate antibody results by age and race/ethnicity. Table showing probability of vaccination given infected, probability of vaccination given uninfected, probability of infection, probability of vaccination, probability of vaccination divided by probability of infection (“risk ratio”), and relative risk ratio with a reference group of White race/ethnicity and the corresponding age.

Age	Race/Ethnicity	parameter	median	lb_2.5	ub_97.5
18-64	Asian	p_vacc_given_inf	0.216	0.008	0.875
18-64	Black	p_vacc_given_inf	0.303	0.012	0.932
18-64	Latinx	p_vacc_given_inf	0.053	0.002	0.261
18-64	White	p_vacc_given_inf	0.125	0.005	0.620
65+	Asian	p_vacc_given_inf	0.233	0.007	0.891
65+	Black	p_vacc_given_inf	0.387	0.015	0.958
65+	Latinx	p_vacc_given_inf	0.270	0.010	0.914
65+	White	p_vacc_given_inf	0.105	0.003	0.688
18-64	Asian	p_vacc_given_uninf	0.099	0.065	0.137
18-64	Black	p_vacc_given_uninf	0.074	0.036	0.124
18-64	Latinx	p_vacc_given_uninf	0.086	0.045	0.141
18-64	White	p_vacc_given_uninf	0.078	0.052	0.108
65+	Asian	p_vacc_given_uninf	0.294	0.245	0.344
65+	Black	p_vacc_given_uninf	0.130	0.054	0.241
65+	Latinx	p_vacc_given_uninf	0.285	0.168	0.420
65+	White	p_vacc_given_uninf	0.316	0.269	0.364
18-64	Asian	p_inf	0.046	0.019	0.086
18-64	Black	p_inf	0.069	0.031	0.124
18-64	Latinx	p_inf	0.291	0.232	0.355
18-64	White	p_inf	0.054	0.028	0.088
65+	Asian	p_inf	0.024	0.005	0.067
65+	Black	p_inf	0.081	0.022	0.184
65+	Latinx	p_inf	0.188	0.090	0.311
65+	White	p_inf	0.046	0.016	0.096
18-64	Asian	p_vacc	0.106	0.072	0.147
18-64	Black	p_vacc	0.093	0.046	0.154
18-64	Latinx	p_vacc	0.080	0.042	0.145
18-64	White	p_vacc	0.083	0.056	0.116
65+	Asian	p_vacc	0.292	0.244	0.343
65+	Black	p_vacc	0.155	0.068	0.270
65+	Latinx	p_vacc	0.290	0.165	0.438
65+	White	p_vacc	0.308	0.260	0.358
18-64	Asian	ratio_p_vacc_p_inf	2.314	1.016	6.304
18-64	Black	ratio_p_vacc_p_inf	1.368	0.510	3.388
18-64	Latinx	ratio_p_vacc_p_inf	0.275	0.134	0.526
18-64	White	ratio_p_vacc_p_inf	1.515	0.754	3.350
65+	Asian	ratio_p_vacc_p_inf	12.220	4.188	55.534
65+	Black	ratio_p_vacc_p_inf	1.912	0.559	7.817
65+	Latinx	ratio_p_vacc_p_inf	1.572	0.655	3.705
65+	White	ratio_p_vacc_p_inf	6.676	2.930	20.444
18-64	Asian	RRR_vs_white_of_that_age	1.571	0.493	4.832
18-64	Black	RRR_vs_white_of_that_age	0.875	0.265	2.883
18-64	Latinx	RRR_vs_white_of_that_age	0.184	0.064	0.495
18-64	White	RRR_vs_white_of_that_age	1.000	1.000	1.000
65+	Asian	RRR_vs_white_of_that_age	1.831	0.405	9.568
65+	Black	RRR_vs_white_of_that_age	0.291	0.060	1.489
65+	Latinx	RRR_vs_white_of_that_age	0.231	0.058	0.788
65+	White	RRR_vs_white_of_that_age	1.000	1.000	1.000

Supplementary Figures

Supplementary Figure 1: Pathways to immunity via infection and vaccination.

Venn diagram showing the pathways to immunity and associated results on Roche and Vitros assays. The blue portion of the Venn diagram shows underlying vaccination and infection states which lead to a positive result on both Roche and Vitros assays. Green portions of the Venn diagram show vaccination and infection states leading to a positive result on the Vitros and negative result on the Roche assay. Samples can either be A) vaccinated and previously infected, B) vaccinated and previously uninfected, C) unvaccinated and previously infected or D) unvaccinated and previously uninfected.

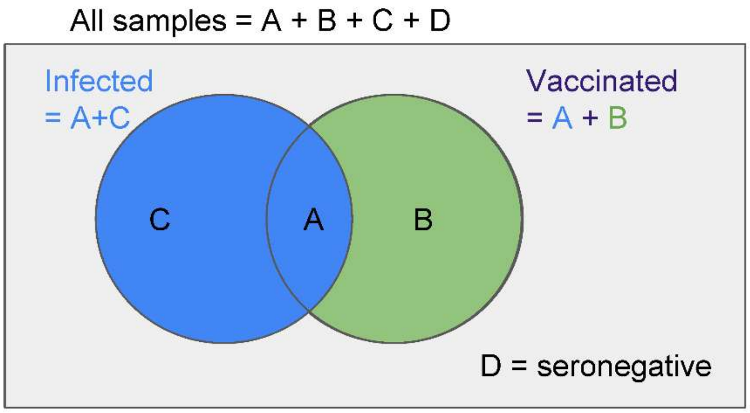

	Vitros	Roche
B	Positive	Negative
A+C	Positive	Positive
D	Negative	Negative

A= Vaccinated and previously infected
B = Vaccinated and not previously infected
C = Not vaccinated and previously infected
D= No immune response (Negative on both assays)

Supplementary Figure 2: Geographic disparities in SARS-CoV-2 infection and vaccination by ZIP code in San Francisco, as of February 2021 (univariate). (A) Adjusted seroprevalence on the Roche assay (reflecting natural infection only). **(B)** Adjusted seroprevalence on the Vitros assay (reflecting both natural infection and/or vaccination). **(C)** The difference in adjusted seroprevalence on the Vitros and Roche assays (reflecting those who were seropositive by vaccination only).

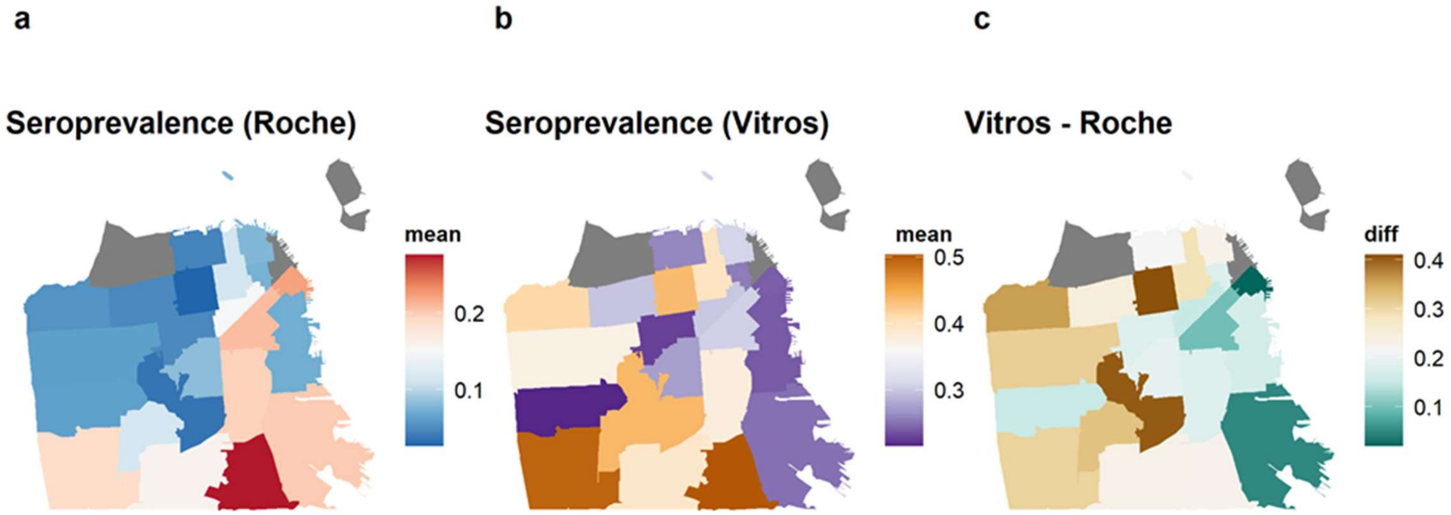

Supplementary Figure 3: Maps showing distributions of the San Francisco population by race/ethnicity and income.

Maps showing a) the proportion of the population which identifies as Hispanic/Latinx by zipcode, b) the proportion of the population which identifies as Black/African American by zipcode, and c) the proportion of the population with a household income below the US census defined poverty line by zipcode. All data come from the 2019 American Community Survey 5 year estimates[1].

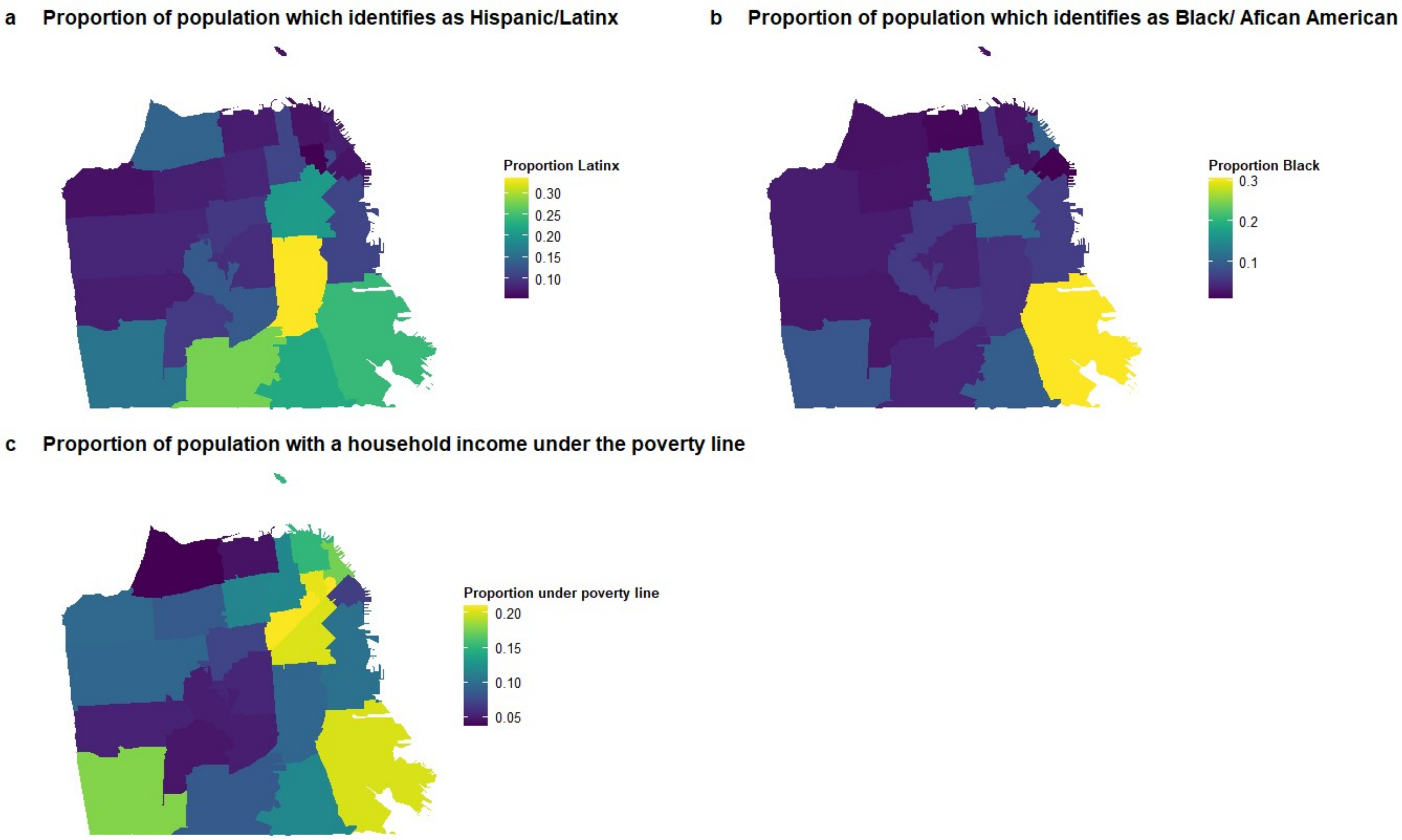

Supplementary Figure 4: Cumulative proportion of individuals who received the first dose of SARS-CoV-2 vaccine by date, age group, and race/ethnicity. Sampling period of this study shown in grey box. Data downloaded from: https://data.sfgov.org/COVID-19/COVID-19-Vaccine-Doses-Given-to-San-Franciscans-by/xjh5-h442.

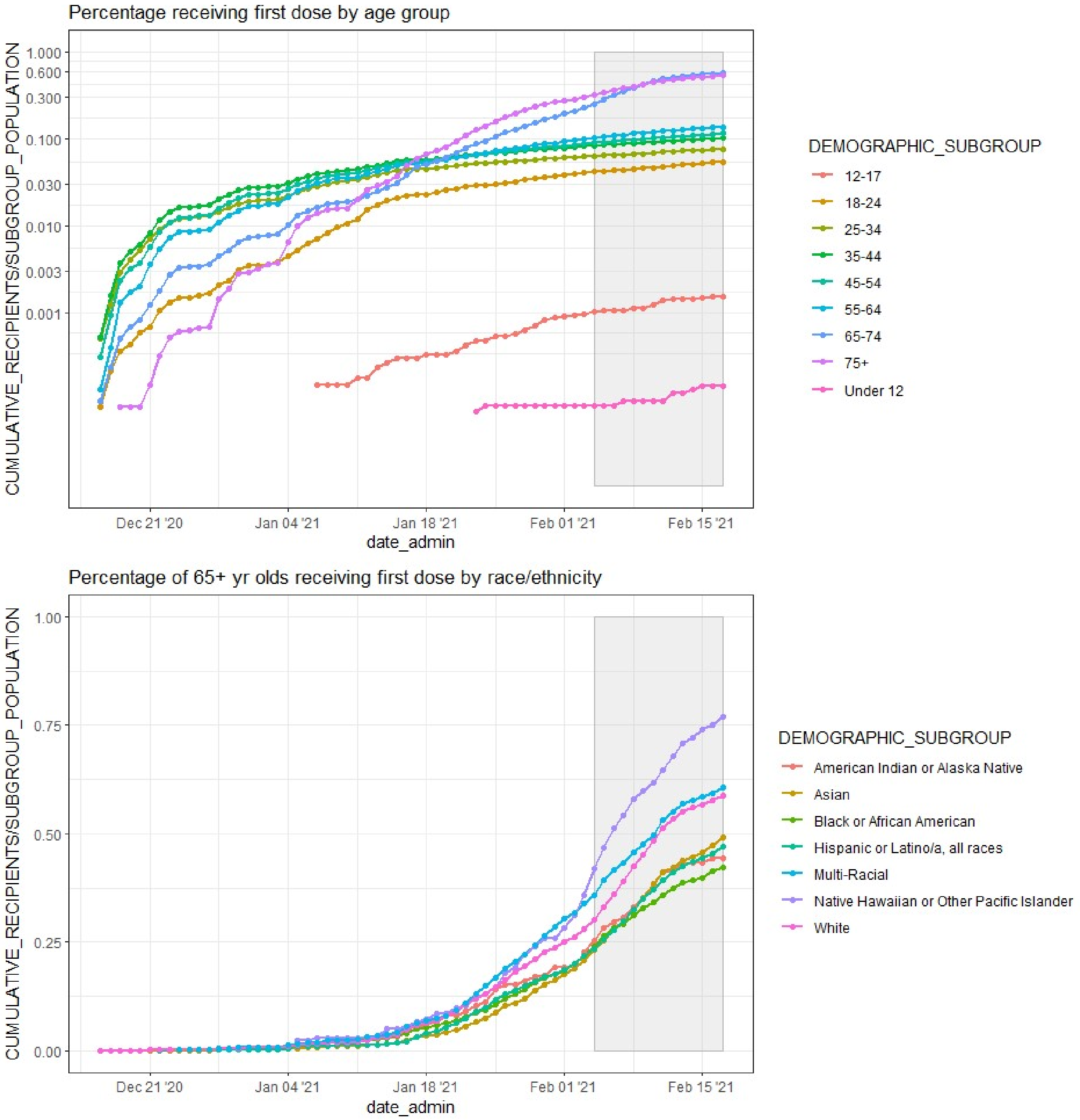

Supplementary Figure 5: Time series of reported SARS-CoV-2 cases and deaths in San Francisco. Sampling period of this study shown in red box. Data downloaded from: https://data.sfgov.org/COVID-19/COVID-19-Cases-Summarized-by-Date-Transmission-and/tvq9-ec9w.

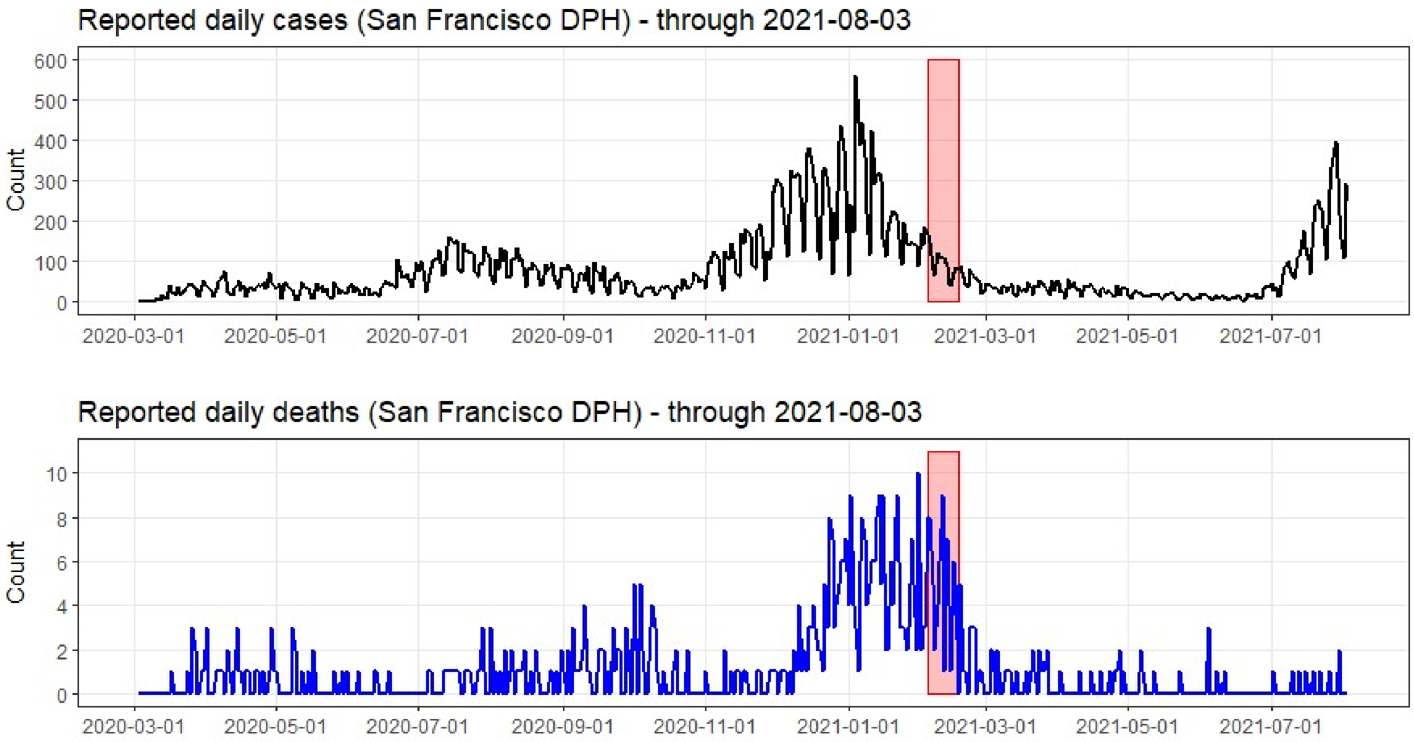

Supplementary References

1. U.S. Census Bureau. 2015-2019 American Community Survey 5-Year Estimates. (2020).
