# Supplementary material for: Using sero-epidemiology to monitor disparities in vaccination and infection with SARS-CoV-2
Source: Nat Commun. 2022 May 4;13:2451. doi: 10.1038/s41467-022-30051-x (PMC9068757; doi:10.1038/s41467-022-30051-x)
Supplement: Supplementary file 3 — Reporting Summary [file 41467_2022_30051_MOESM3_ESM.pdf]

nature portfolio

Corresponding author(s): Routledge, Isobel

Last updated by author(s): Mar 16, 2022

Reporting Summary

Nature Portfolio wishes to improve the reproducibility of the work that we publish. This form provides structure for consistency and transparency in reporting. For further information on Nature Portfolio policies, see our Editorial Policies and the Editorial Policy Checklist.

Statistics

For all statistical analyses, confirm that the following items are present in the figure legend, table legend, main text, or Methods section.

n/a	Confirmed	
☐	☒	The exact sample size (*n*) for each experimental group/condition, given as a discrete number and unit of measurement
☐	☒	A statement on whether measurements were taken from distinct samples or whether the same sample was measured repeatedly
☒	☐	The statistical test(s) used AND whether they are one- or two-sided<br>*Only common tests should be described solely by name; describe more complex techniques in the Methods section.*
☐	☒	A description of all covariates tested
☐	☒	A description of any assumptions or corrections, such as tests of normality and adjustment for multiple comparisons
☐	☒	A full description of the statistical parameters including central tendency (e.g. means) or other basic estimates (e.g. regression coefficient) AND variation (e.g. standard deviation) or associated estimates of uncertainty (e.g. confidence intervals)
☒	☐	For null hypothesis testing, the test statistic (e.g. *F*, *t*, *r*) with confidence intervals, effect sizes, degrees of freedom and *P* value noted<br>*Give P values as exact values whenever suitable.*
☐	☒	For Bayesian analysis, information on the choice of priors and Markov chain Monte Carlo settings
☒	☐	For hierarchical and complex designs, identification of the appropriate level for tests and full reporting of outcomes
☒	☐	Estimates of effect sizes (e.g. Cohen's *d*, Pearson's *r*), indicating how they were calculated

Our web collection on statistics for biologists contains articles on many of the points above.

Software and code

Policy information about availability of computer code

Data collection	Our sampling algorithm used to select samples was written in R version 4.0 and is available at https://github.com/EPPIcenter/scale-it and described in previously published literature (https://doi.org/10.1038/s41467-021-23651-6)
Data analysis	The Stan (version2.21.2) code used to analyse the seroprevalence data to produce estimates of p(vaccinated) and p(infected), stratified by geography and demography are available at https://github.com/EPPIcenter/scale-it-2. The R code and shapefiles to generate Figure 2 was used and described in previously published literature (https://doi.org/10.1038/s41467-021-23651-6) and are available at https://github.com/EPPIcenter/scale-it. The R code to produce Figures 3 and 4 and 5a as well as the Stan/R code to produce the underlying data shown in Figures 3, 4 and 5 are available at https://github.com/EPPIcenter/scale-it-2.

For manuscripts utilizing custom algorithms or software that are central to the research but not yet described in published literature, software must be made available to editors and reviewers. We strongly encourage code deposition in a community repository (e.g. GitHub). See the Nature Portfolio guidelines for submitting code & software for further information.

Data

Policy information about availability of data

All manuscripts must include a data availability statement. This statement should provide the following information, where applicable:

- Accession codes, unique identifiers, or web links for publicly available datasets
- A description of any restrictions on data availability
- For clinical datasets or third party data, please ensure that the statement adheres to our policy

To avoid identifiability of the data and comply with institutional policy around data privacy, we have provided raw data summarized by demographic group (Supplementary Table 3) instead of individual level data. In addition the summary for the raw data results for each assay is available in Supplementary Table 1. The

summary of posterior estimates for p(vaccination) and p(infection) are provided by geography (Supplementary Table 2) and demography (Supplementary Table 3), and by both race and age as a bivariate analysis in Supplementary Table 4. Figure 2a-b summarizes the data described in Table 1 and Supplementary Table 2. Figure 3 uses the data from Supplementary Table 2 and a publicly available shapefile of San Francisco zipcodes (City of San Francisco, SF data, 2021 retrieved from: https://data.sfgov.org/Geographic-Locations-and-Boundaries/Bay-Area-ZIP-Codes/u5j3-svi6). Figure 3 shows the data found in Supplementary Table 2. Figure 4 shows data found in Supplementary Table 3.

Field-specific reporting

Please select the one below that is the best fit for your research. If you are not sure, read the appropriate sections before making your selection.

☒ Life sciences ☐ Behavioural & social sciences ☐ Ecological, evolutionary & environmental sciences

For a reference copy of the document with all sections, see nature.com/documents/nr-reporting-summary-flat.pdf

Life sciences study design

All studies must disclose on these points even when the disclosure is negative.

Sample size

We aimed to collect a total of 1000 samples over two weeks. We determined this sample size based on considerations of both statistical power and feasibility. To estimate seroprevalence with an absolute error of 5% and at Type I error of 5%, and a prior of 20% seroprevalence, a sample size of 246 individuals would need to be tested per age group. We determined that an overall sample size of 1000 samples in total would be sufficient to allow stratification of results by four age groups.

Data exclusions

From the full list of residual serum samples that were available, we restricted our sampling frame to samples from individuals undergoing routine blood testing, to target a population which was more representative of community transmission, rather than oversampling from a population which may be seriously ill. We included patients residing in San Francisco, including those experiencing homelessness. We excluded individuals who were tested for SARS-CoV-2 during the visit when they received their blood draw (except if the test was for routine purposes, such as testing prior to an elective procedure or admittance to the hospital). This was to reduce bias in our sample related to treatment seeking behaviour. We restricted our sample to outpatient and emergency department visits for adults; to have a sample which was more representative of community transmission. For the youngest age group, we included both inpatient and outpatient visits due to small numbers of available samples. We only obtained samples from unique individuals. All of the stated exclusion criteria above were pre-established at the study design stage.

After obtaining the list of eligible samples according to the above criteria, we selected serum samples for the study using a sampling algorithm aimed to ensure an adequate sample size for each of age strata and to maximize geographic representativity. After setting a daily target sample size for our overall population, we divided this equally between four age bins to set a target sample size for each age bin. We also set a target sample size for each zip code which was proportional to its population size. For each zipcode with a larger number of eligible samples than its target size, we kept all samples from age groups with sample sizes below or at their target and obtained a random sample from any age group that had an eligible sample size above the target size.

A total of 1,091 samples were collected using this approach, of which 77 were excluded later due to participation in a separate COVID research study, and a further 15 were later excluded from further analyses as they could not be linked to antibody test results. Of the 999 samples where assay results were available, N = 81 samples were excluded from the bivariate analyses due to missing data in at least one assay, and 3 additional samples were excluded due to positive results on the Roche assay despite negative results on the Vitros assay.

Replication

Code used for the analysis has been made available on GitHub at https://github.com/EPPIcenter/scale-it and https://github.com/EPPIcenter/scale-it-2 and produces reproducible results.

Randomization

Our study was observational so there was no assignment to groups or treatments.When samples were selected following our inclusion/exclusion criteria, samples were selected at random within their stratified groups. Our stratified sampling approach, inclusion/exclusion criteria and adjustment of estimates for assay sensitivity and specificity all assist in controlling for covariates and reducing biases.

Blinding

There was no allocation of groups, as this was an observational study. However samples were only referred to by tube barcode and a unique patient identifier, and re-linked to demographic information once seropositivity/negativity was determined, meaning during both data collection and analysis, researchers were blinded to the demographic information associated with the samples.

Reporting for specific materials, systems and methods

We require information from authors about some types of materials, experimental systems and methods used in many studies. Here, indicate whether each material, system or method listed is relevant to your study. If you are not sure if a list item applies to your research, read the appropriate section before selecting a response.

Materials & experimental systems

n/a	Involved in the study
☐	☒ Antibodies
☒	☐ Eukaryotic cell lines
☒	☐ Palaeontology and archaeology
☒	☐ Animals and other organisms
☐	☒ Human research participants
☒	☐ Clinical data
☒	☐ Dual use research of concern

Methods

n/a	Involved in the study
☒	☐ ChIP-seq
☒	☐ Flow cytometry
☒	☐ MRI-based neuroimaging

Antibodies

Antibodies used	We used 2 commercial serologic assays with US FDA emergency use authorization for this analysis: the Ortho Clinical Diagnostics VITROS Total measuring the total Ig antibody response to the S1 subunit of the SARS-CoV-2 spike (S) protein, and the Roche Elecsys Anti-SARS-CoV-2 assay measuring the total Ig antibody response to the SARS-CoV-2 nucleocapsid (N) protein.
Validation	N/A as no primary antibodies were used - the antibodies were antibodies contained within the samples.

Human research participants

Policy information about studies involving human research participants

Population characteristics	The sample population characteristics are detailed in Table 1 and Figure 2 of the manuscript.By sex the population was N = 509 (50.2 %) female, N = 504 (49.7 %) male and N=1 (0.1%) unknown sex. By age, N= 21 (2.1 %) was aged 0-17, N =157 (15.5%) was aged 18-34, N = 442 (43.60%) was aged 35-64 , and N = 393(38.80 %) was aged 65+, N=1 (0.1%) was unknown. Samples from adults (18+) were all outpatients or emergency room patients within the San Francisco Department of Public Health or University of California San Francisco health networks. For under 18 year olds, both inpatients and outpatients undergoing were used. No genotypic information was available for the samples. We excluded individuals who were tested for SARS-CoV-2 during the visit when they received their blood draw (except if the test was for routine purposes, such as testing prior to an elective procedure or admittance to the hospital). But otherwise we did not exclude or include populations based on any past or current diagnoses.
Recruitment	Samples were obtained as residual sera taken from individuals undergoing routine blood testing, either through the UCSF Health network, or through the San Francisco Department of Public Health/Zuckerberg San Francisco General Hospital network. Therefore our study only captured those served by these hospital networks. However, the networks serve different patient populations because the ZSFGH serves underinsured or uninsured patients.
Ethics oversight	This study received expedited review approval by the UCSF IRB #20-30379 (Serological Surveillance of SARS-CoV-2 in Residual Serum/Plasma Samples). The IRB did not require patient contact or written consent to use residual sera.

Note that full information on the approval of the study protocol must also be provided in the manuscript.
